# A survival analysis of surgically treated incidental low-grade glioma patients

**DOI:** 10.1038/s41598-021-88023-y

**Published:** 2021-04-19

**Authors:** Lingcheng Zeng, Qi Mei, Hua Li, Changshu Ke, Jiasheng Yu, Jian Chen

**Affiliations:** 1 Department of Neurosurgery, Tongji Hospital, Tongji Medical College, Huazhong University of Science and Technology, Wuhan, 430030 Hubei China; 2 Department of Oncology, Tongji Hospital, Tongji Medical College, Huazhong University of Science and Technology, Wuhan, 430030 Hubei China; 3 Department of Pathology, Tongji Hospital, Tongji Medical College, Huazhong University of Science and Technology, Wuhan, 430030 Hubei China

**Keywords:** CNS cancer, Surgical oncology

## Abstract

To evaluate the surgical effect on survival in patients with incidental low-grade glioma (LGG) through comparison between asymptomatic and symptomatic patients. The medical records of surgically treated adult cerebral incidental LGG (iLGG) patients in our department between January 2008 and December 2015 were retrospectively reviewed. The survival of patients was calculated starting from the initial imaging diagnosis. Factors related to progression-free survival (PFS), overall survival (OS) and malignant progression-free survival (MPFS) were statistically analyzed. Seventy-five iLGG patients underwent surgery: 49 in the asymptomatic group, who underwent surgery in the asymptomatic period, and 26 in the symptomatic group, who underwent surgery after the tumor had grown and the patients had developed tumor-related symptoms. Significantly more tumors were initially located adjacent to the functional area in the symptomatic group than in the asymptomatic group (*P* < 0.05), but there was no significant difference in the total resection rate between the two groups. The incidence of postoperative complications (15.4%) and postoperative epilepsy (23.1%) was higher in the symptomatic group than in the asymptomatic group (4.1% and 10.2%, respectively). Multivariate analysis showed that surgical timing, namely, surgery performed before or after symptom occurrence, had no significant effect on PFS, OS or MPFS, while total resection significantly prolonged PFS, OS and MPFS, and the pathology of oligodendroglioma was positively correlated with PFS and OS (*P* < 0.05). Surgical timing for iLGGs should facilitate total resection. If total resection can be achieved, even after symptom occurrence, patients can achieve comparable survival benefits to those treated with surgery in the asymptomatic phase.

## Introduction

With the increasing application of head MRI, a growing number of WHO grade II gliomas (low-grade gliomas, LGGs) have been incidentally found, but some patients do not manifest any tumor-related symptoms. Such incidental LGGs (iLGGs) are uncommon and account for only approximately 3.8% to 10.4% of all LGGs^1–4^. Surgical resection is the primary treatment. Since patients manifest no tumor-related symptoms, there are still different views on the timing of surgery, such as whether to wait until the patient develops tumor-related symptoms^5–7^. The analysis of survival-related factors is helpful for surgical decisions. Some retrospective studies have shown that the overall survival (OS) of iLGG patients treated with surgery is better than that of symptomatic LGG patients^2–4^.

LGGs are characterized by slow growth; thus, the progression of asymptomatic iLGG to symptomatic LGG may last for years or even decades^5,8^, so the calculation of survival time for symptomatic LGG, which begins when patients are asymptomatic at the first imaging diagnosis, differs from that which begins when patients are symptomatic at the first surgery, and such differences may affect survival analyses^5^. All symptomatic LGGs develop from asymptomatic LGGs, so the calculation of survival time, which begins from the first imaging diagnosis at the asymptomatic phase, can accurately reflect their natural history. To minimize the impact of such bias on survival analyses, we retrospectively reviewed the medical data of surgically treated iLGG patients in our department and calculated survival from the time of the initial imaging diagnosis. Then, we compared the patients who underwent surgical treatment in the asymptomatic period with those who underwent surgical treatment when they manifested tumor-related symptoms and assessed the prognostic factors. Different from previous studies^2–4^, due to the similar starting point in calculating the survival time, this study can more accurately evaluate the effect of factor of symptom on patient survival, which can aid in decisions on optimal surgical timing for iLGG patients.

## Materials and methods

### Patients

This study was conducted retrospectively using the data obtained for clinical purposes. Proper documents including the approval of all experimental protocols, the official waiver of ethical approval and informed consent were obtained from the Human Investigation Committee (IRB) of Huazhong University of Science and Technology for this study. All methods were performed in accordance with the relevant guidelines and regulations. The medical records of adult cerebral iLGG patients (≥ 18 years old) admitted to the Department of Neurosurgery, Tongji Hospital affiliated with Tongji Medical College of Huazhong University of Science and Technology between January 2008 and December 2015 were retrospectively reviewed. All patients were surgically treated and pathologically confirmed. Pathological paraffin specimens were collected and further examined for IDH1 R132 and IDH2 R172 mutations, the 1p/19q codeletion and the CDKN2A/B homozygous deletion. In addition to the classical microscopical features of WHO grade II LGG, diffuse astrocytoma was diagnosed upon detection of the molecular characteristics of an IDH mutation without 1p/19q codeletion or the CDKN2A/B homozygous deletion, and oligodendroglioma was diagnosed upon detection of an IDH mutation with 1p/19q codeletion. Tumors with IDH wild type or CDKN2A/B homozygous deletion were not included in this study, as the Consortium to Inform Molecular and Practical Approaches to CNS Tumor Taxonomy (cIMPACT-NOW) suggests that most of them should be classified as grade IV rather than grade II^9–11^.

Patient data, including age, sex, preoperative Karnofsky performance score (KPS), and preoperative MRI data, including tumor location and size, were collected. Tumor size was assessed by recording the maximum diameters of the axial, coronal and sagittal sections of the tumor, and changes in the maximum diameters were evaluated during follow-up to assess tumor progression. To assess the annual growth rate of the tumor, the change in the maximum diameter of the section with the fastest growth was calculated, divided by the month of follow-up, and then multiplied by 12 months. The extent of resection was determined with T2 FLAIR MR imaging at 1–3 months after surgery and classified as total resection, subtotal resection (90–99%), partial resection (50–89%) and biopsy (< 50%).

After 2010, when resecting tumors adjacent to the functional area, including the inferior posterior frontal gyrus in the dominant hemisphere and the anterior central gyrus, intraoperative assistant techniques such as awake surgery, intraoperative electrophysiological mapping of the functional area and intraoperative ultrasound were employed. A tumor location adjacent to the functional area was defined according to a distance between the tumor border and the brain functional area of less than 1 cm as measured on axial, coronal or sagittal sections of MRI images; otherwise, the location was defined as away from the functional area. Postoperative complications, including functional disorders, epilepsy and KPS score at 3 to 6 months after surgery, and postoperative adjuvant therapy of radiotherapy plus chemotherapy, were recorded. Because procarbazine is not available in China, adjuvant therapy according to the Stupp protocol (radiotherapy plus concomitant and adjuvant temozolomide) was suggested to patients with residual tumors after surgery or aged > 40 years at 1–3 months postoperatively. Tumor recurrence was defined as tumor reappearance or enlargement of a residual tumor on MRI, which was performed every 6 months to 1 year after surgery. Malignant progression was defined as signal changes on the enhanced MR image or an increased grade of malignancy confirmed by histopathology after a reoperation.

### Surgical timing

Surgical timing was classified as two types: (1) surgery before symptom occurrence: surgery was performed after tumor enlargement on MRI imaging during follow-up or before any change in tumor size and signal was noted if the patient had a strong desire for surgery and not enough follow-up imaging was available to evaluate tumor growth; and (2) surgery after symptom occurrence during follow-up: tumor enlargement and symptom occurrence were indicated during follow-up.

### Assessment of progression-free survival (PFS), OS and malignant progression-free survival (MPFS)

The follow-up deadline was December 31, 2019. PFS was defined as the time from the initial imaging diagnosis to evidence of tumor enlargement or recurrence on follow-up MRI after surgery. OS was defined as the time from the initial imaging diagnosis to death. MPFS was defined as the time from the initial imaging diagnosis to evidence of contrast enhancement on follow-up MRI or higher-grade histopathology from subsequent surgery.

### Statistical analysis

SPSS 25.0 software (IBM, New York, USA) was used for statistical analysis. A chi-square test was used to compare the categorical variables between groups. A t test was used to compare the continuous variables between groups. Log-rank univariate analysis was used to identify the PFS-, OS- and MPFS-related factors, and Kaplan–Meier survival curves were drawn. The above statistically significant factors were further analyzed by Cox regression multivariate analysis. A *P* value less than 0.05 was considered statistically significant.

### Ethics approval

This research study was conducted retrospectively from data obtained for clinical purposes. We consulted extensively with the Human Investigation Committee (IRB) of Huazhong University of Science and Technology who determined that our study did not need ethical approval. An IRB official waiver of ethical approval was granted from the IRB of Huazhong University of Science and Technology.

## Results

### Clinical characteristics

Eighty-five adult iLGGs patients were surgically treated between January 2008 and December 2015; 10 patients were lost to follow-up due to incomplete information that was required for the study, so 75 iLGG patients were ultimately included. The reasons for the discovery were as follows: 35 patients, physical examination; 14 patients, head trauma; 10 patients, dizziness; 6 patients, sinusitis; 5 patients, trigeminal neuralgia; and 5 patients, nonspecific headache. The preoperative KPS of all patients was more than 90. There were 33 males (44%) and 42 females (56%), with a median age of 38 years ranging from 21 to 53 years. Thirty-four lesions (45.3%) were located in the frontal lobe, 16 lesions (21.3%) were in the temporal lobe, 10 lesions (13.3%) were in the parietal lobe, 8 lesions (10.7%) were in the occipital lobe, and 7 lesions (9.3%) were in the insular lobe. The mean tumor diameter was 2.5 ± 0.7 cm and ranged from 1.3 to 4.2 cm. Total resection was achieved in 59 patients (78.7%), subtotal resection in 13 patients (17.3%), and partial resection in 3 patients (4%). Diffuse astrocytoma occurred in 44 patients (58.7%). Low-grade oligodendroglioma occurred in 31 patients (41.3%).

Forty-nine patients were surgically treated at the asymptomatic phase of the tumor and were assigned to the asymptomatic group: 16 patients underwent surgery before any change in tumor size and signal, and 33 patients underwent surgery after tumor enlargement on MRI imaging during follow-up. The remaining 26 patients who underwent surgery after tumor enlargement and symptom onset were assigned to the symptomatic group. Detailed symptoms associated with the tumor sites are listed in Table 1. The clinical characteristics of the asymptomatic group and the symptomatic group were compared, and no significant differences were found in sex, age, tumor size, extent of resection, pathological diagnosis or postoperative adjuvant chemo-radiotherapy (CRT) (*P* > 0.05, Table 2). Significantly more tumors were located adjacent to the functional area, including the inferior posterior frontal gyrus in the dominant hemisphere (2 cases), anterior central gyrus (8 cases), left superior posterior temporal gyrus (2 cases), posterior central gyrus (2 cases) and basal ganglia region (2 cases), in the symptomatic group than in the asymptomatic group (*P* < 0.05, Table 2).

**Table 1:** The detailed symptoms associated with tumor sites in the symptomatic group.

Tumor site	Symptom (n)
Frontal lobe	Jacksonian seizure (8)
	Generalized tonic–clonic seizure and increased headaches (3)
	Contralateral limb weakness (2)
Temporal lobe	Sensory aphasia (2)
	Psychomotor seizure (1)
Insular lobe	Psychomotor seizure (2)
	Generalized tonic–clonic seizure (2)
Parietal lobe	Contralateral limb numbness (2)
	Focal sensory seizure (1)
Occipital lobe	Visual hallucination and blurring (2)
	Increased headaches (1)

**Table 2:** The clinical characteristics of incidental low grade glioma classified into asymptomatic or symptomatic group.

	Asymptomatic group(n = 49)	Symptomatic group(n = 26)	*P* value^a^
**Sex**			0.446
Male	20	13	
Female	29	13	
**Age (year)**			0.129^b^
20–29	7	2	
30–39	18	16	
40–49	23	8	
50–59	1	0	
**Tumor site**			0.001^c^
Frontal lobe	21	13	
Temporal lobe	13	3	
Insular lobe	3	4	
Parietal lobe	7	3	
Occipital lobe	5	3	
Adjacent to functional area	11	16	
Away from functional area	38	10	
**Tumor diameter (cm)**			0.196
≤ 2	13	3	
2–3	29	16	
> 3	7	7	
**Extent of resection**			0.146^d^
Total resection	41	18	
Subtotal resection	6	7	
Partial resection	2	1	
Biopsy	0	0	
**Pathology**			0.267
Diffuse astrocytoma	31	13	
Oligodendrocytoma	18	13	
**Radiotherapy + temozolomide**			0.842
Yes	14	8	
No	35	18	

^a^*P* value based on Chi-squared test.

^b^The group of “age ≤ 39” was compared with the group of “age > 40”.

^c^The group of tumor site “adjacent to functional area” was compared with the group of “away from functional area”.

^d^The group of “total resection” was compared with the group of non-total resection.

### Assessment of tumor growth

Fifty-nine patients (33 asymptomatic patients and 26 symptomatic patients) received regular MRI examinations during follow-up before surgery, and the annual rate of tumor growth was evaluated. The mean preoperative follow-up periods were 30 ± 10 months (range 12–60 months) in the whole group, 25 ± 7 months (range 12–38 months) in the asymptomatic group and 35 ± 10 months (range 18–60 months) in the symptomatic group. The symptomatic group was preoperatively followed up significantly longer than the asymptomatic group (*P* < 0.001). The average growth rate of tumors in the whole group was 2.7 ± 0.9 mm/year (1–5 mm/year), while that in the asymptomatic group was 2.9 ± 0.9 mm/year (1–5 mm/year), and that in the symptomatic group was 2.5 ± 0.7 mm/year (1–4 mm/year); there was no significant differences between the two groups (*P* = 0.0827).

### Assessment of clinical outcomes

Surgical complications occurred in 2 patients (4.1%) in the asymptomatic group, including 1 case of epidural hematoma and 1 case of brain hematoma that required surgery. No dysfunction after surgery occurred. Five patients in this group developed postoperative epilepsy (5/49, 10.2%). The KPS of all patients in this group was ≥ 90 at 3–6 months postoperatively.

Surgical complications occurred in 4 patients (15.4%) in the symptomatic group, including 2 cases of mild hemiplegia, 1 case of mild aphasia and 1 case of wound infection. For the 2 patients with postsurgical hemiplegia, the tumors were located near the precentral gyrus. For the patient with postsurgical aphasia, the tumor was located near the inferior posterior frontal gyrus in the dominant hemisphere. In all three patients with complications, no awake surgery or intraoperative electrophysiological mapping was employed during surgery before 2010, but since 2010, due to the application of the two techniques, no complications occurred in the motor or language area of the remaining 7 patients. Despite a high incidence of preoperative epilepsy (17/26, 65.4%) in the symptomatic group, the incidence of epilepsy decreased (6/26, 23.1%) after surgical treatment. The other preoperative symptoms such as limb weakness or numbness, sensory aphasia, headaches, visual hallucinations and blurring resolved gradually after surgery. The KPS of all patients was ≥ 90 at 3–6 months postsurgery.

In general, the incidence of complications and epilepsy after surgery was higher in the symptomatic group than in the asymptomatic group, while all patients returned to normal activities at 3–6 months postoperatively.

### Univariate analyses of the factors related to PFS, OS and MPFS

**Figure 1:**
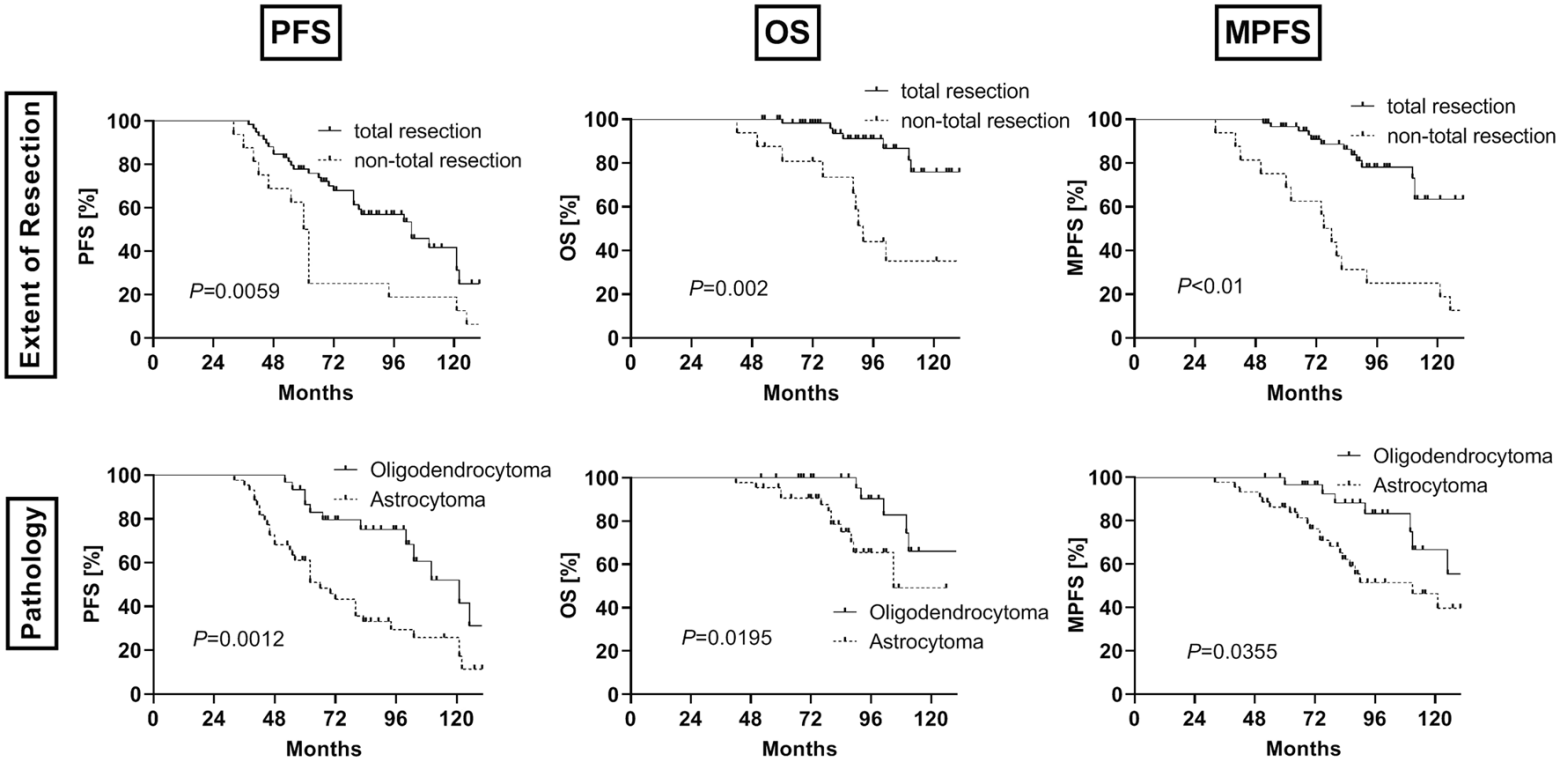
Kaplan–Meier curves showed the PFS (first column), OS (second column) and MPFS (third column) associated with the factors of extent of resection (first row) and tumor pathology (second row).

**Figure 2:**
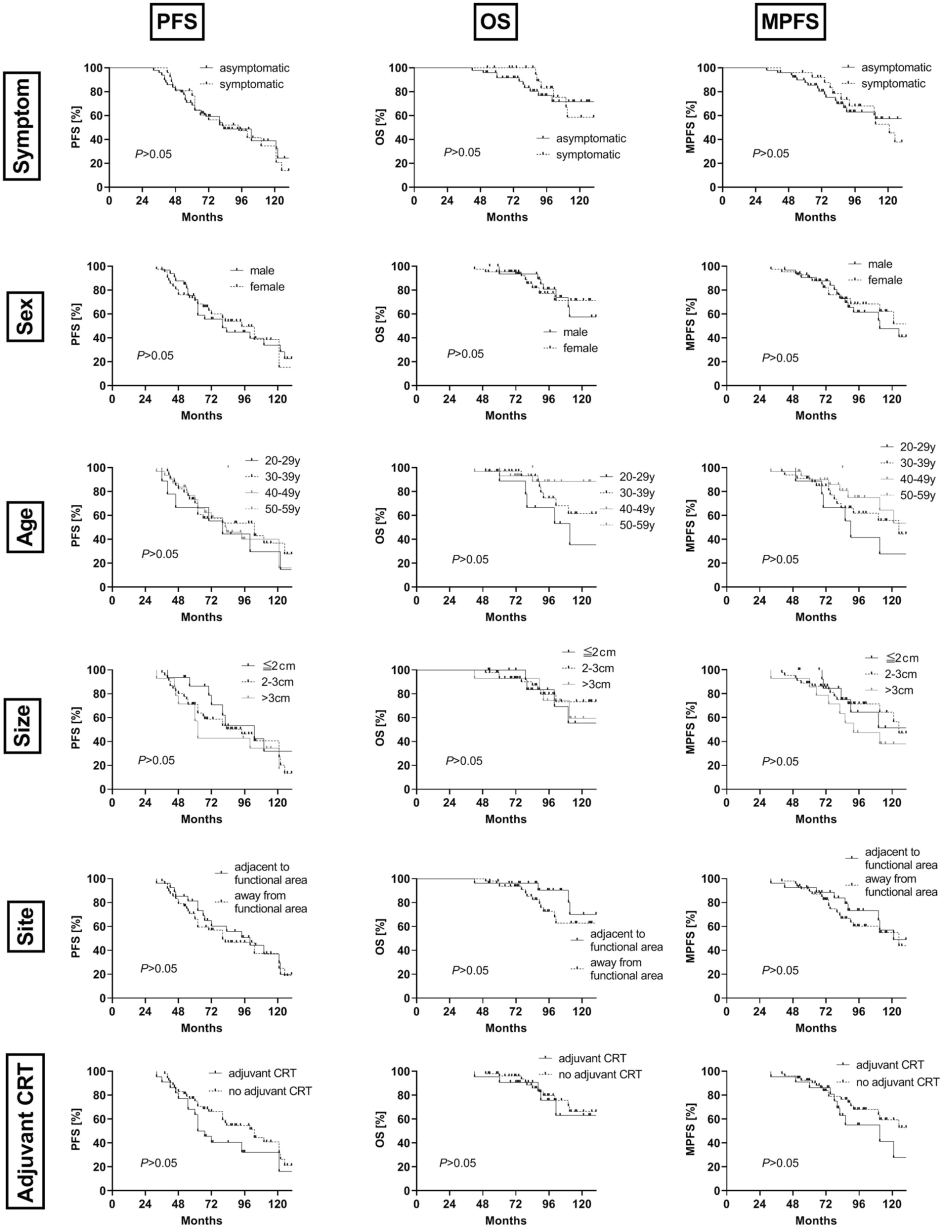
Kaplan–Meier curves showed the PFS (first column), OS (second column) and MPFS (third column) analyzed by the factors of symptom (first row), sex (second row),age (third row), tumor size (fourth row), tumor site (fifth row) and adjuvant chemo-radiotherapy (CRT) (sixth row).

Total resection and pathology of oligodendroglioma were positively correlated with PFS, OS and MPFS in the whole group (all *P* < 0.05, Fig. 1), while the other factors, including symptoms, sex, age, tumor size, tumor site (adjacent to the functional area) and adjuvant CRT, had no significant correlation with patient survival (*P* > 0.05, Fig. 2).

The PFS rates at 5 and 10 years in the total resection group were 78% and 31%, and those in the nontotal resection group at 5 and 10 years were 50% and 13%. The PFS rates at 5 and 10 years in the oligodendroglioma group were 87% and 42%, and those in the astrocytoma group were 61% and 17%.

The OS rates at 5 and 10 years in the total resection group were 98% and 76%, and those in the nontotal resection group at 5 and 10 years were 81% and 35%. The OS rates at 5 and 10 years in the oligodendroglioma group were 100% and 66%, and those in the astrocytoma group were 91% and 49%.

The MPFS rates at 5 and 10 years in the total resection group were 97% and 63%, and those in the nontotal resection group at 5 and 10 years were 69% and 19%. The MPFS rates at 5 and 10 years in the oligodendroglioma group were 97% and 67%, and those in the astrocytoma group were 86% and 40%.

### Multivariate analyses of the factors related to PFS, OS and MPFS

The factors total resection and pathology of oligodendroglioma were further analyzed in the whole group by multivariate Cox regression analysis. The results showed that total resection and pathology of oligodendroglioma were still significantly positively correlated with PFS and OS (*P* < 0.05, Table 3), and total resection was significantly positively correlated with MPFS (*P* < 0.05, Table 3). Since the survival of oligodendroglioma patients was significantly better than that of diffuse astrocytoma patients, a subgroup analysis separating the oligodendroglioma group from the astrocytoma group was further performed, and the factors total resection and postoperative adjuvant CRT were included in the COX regression analysis. The results showed that in either the oligodendroglioma group or the astrocytoma group, total resection was still significantly positively correlated with patients' PFS, OS and MPFS (*P* < 0.05, Table 4), while no significant correlation between adjuvant CRT and patients’ survival could be found (*P* > 0.05, Table 4).

**Table 3:** Cox regression analysis of factors related to PFS, OS and MPFS in the Whole group.

	Total resection	Oligodendrocytoma
**PFS**		
HR	4.507	3.378
95% CI	2.308–8.803	1.712–6.667
*P*	< 0.001	< 0.001
**OS**		
HR	8.286	2.045
95% CI	3.739–18.364	0.001–2.195
*P*	< 0.001	0.002
**MPFS**		
HR	7.006	1.499
95% CI	2.643–18.570	0.908–2.475
*P*	< 0.001	0.113

*HR* hazard ratio, *CI* confidential interval.

**Table 4:** Cox regression analysis of factors related to PFS, OS and MPFS in the sub-group separating oligodendrocytoma from astrocytoma.

	Oligodendrocytoma (n = 31)		Astrocytoma (n = 44)	
	Total resection (n = 24)	Adjuvant CRT (n = 10)	Total resection (n = 35)	Adjuvant CRT (n = 12)
**PFS**				
HR	4.266	0.500	3.341	0.700
95% CI	1.387–13.119	0.135–1.852	1.308–8.533	0.304–1.615
*P*	0.011	0.299	0.012	0.404
**OS**				
HR	6.605	0.341	10.239	3.048
95% CI	1.100–33.426	0.053–2.204	2.717–38.584	0.692–13.415
*P*	0.038	0.259	0.001	0.141
**MPFS**				
HR	11.439	0.176	9.809	1.858
95% CI	1.877–69.726	0.026–1.212	3.215–29.931	0.611–5.646
*P*	0.008	0.078	< 0.001	0.275

*HR* hazard ratio, *CI* confidential interval, *CRT* chemo-radiotherapy.

## Discussion

In this study, through a dynamic comparison of the maximum tumor diameters, we found that iLGGs showed slow, continuous growth, with an annual average growth rate of 2.7 ± 1.0 mm that fluctuated between 1 and 5 mm, which was consistent with the studies of other scholars^2,12,13^. According to growth features, if the lesion size shows a growth rate of several centimeters per year, it is often believed that the lesion is highly malignant. If the lesion size shrinks over years of observation, the lesion should not be a glioma, and no surgery is required. However, patients with no obvious change in size in the first few years need to be followed up for a longer period because a small number of LGGs grow extremely slowly and may suddenly develop malignant progression^14,15^.

This study also showed that iLGGs were characterized by a small initial size, with an average maximum diameter of 2.5 cm that fluctuated between 1.3 and 4.0 cm. Moreover, due to slow tumor growth, it often took years for a tumor to develop symptoms from an incidental discovery. In this study, from the first imaging diagnosis to the onset of symptoms, an average preoperative follow-up of 35 ± 10 months was required. Pallud et al. reported a median follow-up period of 48 months before the onset of tumor-related symptoms^2^, and Gui et al. reported a period of 79.7 months^12^. The difference in the reported follow-up time before the onset of symptoms between studies may be ascribed to the different time points of tumor discovery. The tumor might have been detected by imaging even earlier, but it could not be detected without an examination^8,16^. The period from the generation of an LGG to the occurrence of tumor-related symptoms has been referred to as the silent phase^8^. Pallud et al. attempted to assess the duration of this silent phase. They retrospectively studied the MRI data of 148 patients who underwent at least three MRI examinations during more than 6 months of preoperative follow-up, calculated the initial tumor growth rate and then estimated the silent phase with the initial tumor volume/initial growth rate. They determined that the average silent phase was approximately 14.0 ± 7.8 years, and the median silent phase was approximately 11.6 years, fluctuating between 1.6 and 39.4 years^8^.

Therefore, when comparing the survival of patients with incidental asymptomatic LGG with that of patients with symptomatic LGG, if the calculation of survival of symptomatic LGG patients started from the time of symptom occurrence, ignoring the silent phase of sometimes more than 10 years, the survival analysis might be biased. To take into account the influence of the silent phase on the survival analysis, we compared the patients with iLGG who underwent surgical treatment before symptom occurrence with the patients who underwent surgical treatment after symptom occurrence during follow-up. Moreover, survival was calculated from the first imaging diagnosis when no tumor-related symptoms occurred. In this way, the study showed that the preoperative follow-up period of the symptomatic group was significantly longer than that of the asymptomatic group from the initial imaging diagnosis to surgical treatment, and the omission of this period should affect the survival analysis. Thus, different from previous studies^2–4^, this study showed that surgical timing, namely, whether surgery was performed before or after symptom occurrence, had no significant effects on OS, PFS or MPFS of the two patient groups.

In this study, total resection was a significant factor that positively correlated with OS, PFS and MPFS in both groups. Accordingly, iLGGs should be removed to the largest extent possible rather than simply for the purpose of diagnosis. iLGGs are usually small in size and show no enhancement on MRI; thus, little bleeding will normally occur during tumor resection, which facilitates surgical resection. Although the tumors in the symptomatic group in this study showed the characteristics of being close to the functional area, the majority of patients also obtained total resection with functional protection, and the total resection rate was not significantly different from that in the asymptomatic group, which could also explain why the symptom factor was irrelevant to patient survival. The high total resection rate of lesions adjacent to the functional area was due to the application of increasingly effective intraoperative assistant techniques, such as awake surgery, intraoperative electrophysiological mapping of the functional area and intraoperative ultrasound.

In our practice, we defined a tumor border less than 1 cm from the functional area as measured on preoperative MRI images as a tumor location adjacent to the functional area; larger separations were defined as a tumor location away from the functional area, because no functional disorders after surgery were observed in tumors located more than 1 cm from the functional area in this study. According to this criterion, significantly more tumors were located adjacent to the functional area in the symptomatic group than in the asymptomatic group, and postsurgical functional disorders were only observed in the symptomatic group. In addition, due to the high incidence of epilepsy in the symptomatic group before surgery, despite the decreased incidence of epilepsy after surgery, the incidence of epilepsy after surgery was still higher than that in the asymptomatic group. Therefore, from the perspective of postoperative quality of life, it is reasonable to consider surgical resection before symptom occurrence.

According to the results of this study, the surgical timing for iLGGs should facilitate total resection. In clinical practice, it seems that the achievement of total resection in the asymptomatic phase is much easier than that in the symptomatic phase. As previously described, although the total resection rate of tumors near functional areas was comparable to that of tumors away from functional areas, the requirements for surgical techniques and auxiliary equipment were higher. For tumors away from functional areas, their size is much larger when symptoms develop; thus, the surgical difficulty is also increased. It often takes years for iLGGs developing symptoms, and some LGGs may undergo malignant transformation, making surgery more difficult^14,15^.

This study has some limitations. Firstly, due to the low incidence of iLGGs, although 7 years of cases were collected, the case number in this study was still limited. In addition, some historical cases could not be analyzed due to incomplete information that was required for this study, and thus, these cases had to be excluded. Finally, the effects of CRT on survival were biased to some extent. The CRT effect on patient survival was statistically analyzed in the whole group; however, the postsurgical CRT was mainly recommended to patients with risk factors such as age > 40 years and/or residual tumor after surgery, which resulted in selection bias. Due to the limited case number of iLGG patients with risk factors, the effect of adjuvant CRT could not be statistically analyzed in this subgroup. Because some patients with risk factors refused treatment and some patients without risk factors accepted treatment due to the individual experience with their oncologist, selection bias was weakened but still existed in the statistical analysis of the whole group. In our study, despite the validity of adjuvant procarbazine, lomustine, and vincristine (PCV) chemotherapy demonstrated in the phase III RTOG 9802 trial, due to the unavailability of procarbazine in China, temozolomide was adopted as the postoperative chemotherapy regimen. Some studies have reported the effects of adjuvant temozolomide on survival of LGG patients, but their results differed^17–19^. Although this study found no significant difference in the survival of iLGG patients treated with the Stupp protocol after surgery compared with those who did not receive chemoradiotherapy, due to the limitations and biases present in this retrospective study, the real effect of the Stupp protocol still needs to be further evaluated in an RCT in the future^20^.

## Conclusion

iLGG is a disease with low malignant potential and slow progression. If the calculation of survival of LGG patients takes into account the silent phase of the disease, then surgical timing (namely, whether surgery was performed before or after symptom occurrence) has no significant effect on patients’ survival, but other factors (namely, total resection and the pathology of oligodendroglioma) are significantly positively correlated with PFS and OS. Total resection can also significantly prolong MPFS. Thus, in the clinical practice, if an initial imaging-diagnosed iLGG shows slow growth behavior during follow-up, the diagnosis of LGG should be highly suspected, and surgical planning should be performed. The surgical timing of iLGGs should facilitate total resection.

## Data Availability

The datasets generated during and/or analysed during the current study are available from the corresponding author on reasonable request.

## Code availability

The software application analysed during the current study are available from the corresponding author on reasonable request.

## References

[1] Onizuka M, Suyama K, Shibayama A, Hiura T, Horie N (2001). Asymptomatic brain tumor detected at brain check-up. Neurol. Med. Chir. (Tokyo).

[2] Pallud J, Fontaine D, Duffau H, Mandonnet E, Sanai N (2010). Natural history of incidental World Health Organization grade II gliomas. Ann. Neurol..

[3] Potts MB, Smith JS, Molinaro AM, Berger MS (2012). Natural history and surgical management of incidentally discovered low-grade gliomas. J. Neurosurg..

[4] Zhang ZY, Chan AK, Ng HK, Ding XJ, Li YX (2014). Surgically treated incidentally discovered low-grade gliomas are mostly IDH mutated and 1p19q co-deleted with favorable prognosis. Int. J. Clin. Exp. Pathol..

[5] Recht LD, Lew R, Smith TW (1992). Suspected low-grade glioma: Is deferring treatment safe?. Ann. Neurol..

[6] Wijnenga MMJ, Mattni T, French PJ, Rutten GJ, Leenstra S (2017). Does early resection of presumed low-grade glioma improve survival? A clinical perspective. J. Neurooncol..

[7] Lima GL, Zanello M, Mandonnet E, Taillandier L, Pallud J (2016). Incidental diffuse low-grade gliomas: From early detection to preventive neuro-oncological surgery. Neurosurg. Rev..

[8] Pallud J, Capelle L, Taillandier L, Badoual M, Duffau H (2013). The silent phase of diffuse low-grade gliomas. Is it when we missed the action?. Acta Neurochir. (Wien).

[9] Brat DJ, Aldape K, Colman H, Figrarella-Branger D, Fuller GN (2020). cIMPACT-NOW update 5: Recommended grading criteria and terminologies for IDH-mutant astrocytomas. Acta Neuropathol..

[10] Brat DJ, Aldape K, Colman H, Holland EC, Louis DN (2018). cIMPACT-NOW update 3: Recommended diagnostic criteria for "Diffuse astrocytic glioma, IDH-wildtype, with molecular features of glioblastoma, WHO grade IV". Acta Neuropathol..

[11] Shirahata M, Ono T, Stichel D, Schrimpf D, Reuss DE (2018). Novel, improved grading system(s) for IDH-mutant astrocytic gliomas. Acta Neuropathol..

[12] Gui C, Kosteniuk SE, Lau JC, Megyesi JF (2018). Tumor growth dynamics in serially-imaged low-grade glioma patients. J. Neurooncol..

[13] Opoku-Darko M, Eagles ME, Cadieux M, Isaacs AM, Kelly JJP (2019). Natural history and growth patterns of incidentally discovered diffusely infiltrating low-grade gliomas: A volumetric study. World Neurosurg..

[14] Shah AH, Madhavan K, Heros D, Raper DM, Iorgulescu JB (2011). The management of incidental low-grade gliomas using magnetic resonance imaging: Systematic review and optimal treatment paradigm. Neurosurg. Focus.

[15] Cochereau J, Herbet G, Rigau V, Duffau H (2016). Acute progression of untreated incidental WHO Grade II glioma to glioblastoma in an asymptomatic patient. J. Neurosurg..

[16] Duffau H, Pallud J, Mandonnet E (2011). Evidence for the genesis of WHO grade II glioma in an asymptomatic young adult using repeated MRIs. Acta Neurochir. (Wien).

[17] Baumert BG, Hegi ME, van den Bent MJ, von Deimling A, Gorlia T (2016). Temozolomide chemotherapy versus radiotherapy in high-risk low-grade glioma (EORTC 22033–26033): A randomised, open-label, phase 3 intergroup study. Lancet Oncol..

[18] Paľa A, Coburger J, Scherer M, Ahmeti H, Roder C (2020). To treat or not to treat? A retrospective multicenter assessment of survival in patients with IDH-mutant low-grade glioma based on adjuvant treatment. J. Neurosurg..

[19] Wang J, Yan L, Ai P, He Y, Guan H (2020). Observation versus radiotherapy with or without temozolomide in postoperative WHO grade II high-risk low-grade glioma: A retrospective cohort study. Neurosurg. Rev..

[20] Wang J, Wang Y, He Y, Guan H, He L (2019). Radiotherapy versus radiotherapy combined with temozolomide in high-risk low-grade gliomas after surgery: Study protocol for a randomized controlled clinical trial. Trials.

